# Mitochondrial superoxide in osteocytes perturbs canalicular networks in the setting of age-related osteoporosis

**DOI:** 10.1038/srep09148

**Published:** 2015-03-16

**Authors:** Keiji Kobayashi, Hidetoshi Nojiri, Yoshitomo Saita, Daichi Morikawa, Yusuke Ozawa, Kenji Watanabe, Masato Koike, Yoshinori Asou, Takuji Shirasawa, Koutaro Yokote, Kazuo Kaneko, Takahiko Shimizu

**Affiliations:** 1 Department of Advanced Aging Medicine, Chiba University Graduate School of Medicine, Chiba, Japan; 2 Department of Medicine, Chiba University Graduate School of Medicine, Chiba, Japan; 3 Department of Orthopaedics, Juntendo University Graduate School of Medicine, Tokyo, Japan; 4 Department of Aging Control Medicine, Juntendo University Graduate School of Medicine, Tokyo, Japan; 5 Department of Orthopedic Surgery, Tokyo Medical and Dental University, Tokyo, Japan

## Abstract

Osteocytes are major bone cells that play a crucial role in maintaining the quality of and healing damage to bone tissue. The number of living osteocytes and canalicular networks declines in an age-dependent manner. However, the pathological effects of mitochondrial redox imbalances on osteocytes and bone metabolism have not been fully elucidated. We generated mice lacking mitochondrial superoxide dismutase 2 (*Sod2*) in osteocytes. Like an aged bone, *Sod2* depletion in the osteocytes positively enhanced the production of cellular superoxide *in vivo*. A bone morphological analysis demonstrated that the *Sod2*-deficient femurs showed remarkable bone loss in an age-dependent manner. Interestingly, *Sod2* loss induced markedly disorganized osteocytic canalicular networks and decreased the number of live osteocytes. Furthermore, *Sod2* deficiency significantly suppressed bone formation and increased bone resorption concomitant with the upregulation of sclerostin and receptor activator of NF-κB ligand (RANKL). *In vitro* experiments also revealed that treatment with paraquat, a superoxide inducer in mitochondria, promoted the RANKL expression via, in part, ERK phosphorylation. These findings demonstrate that the mitochondrial superoxide induced in osteocytes by *Sod2* ablation causes age-related bone loss due to the impairment of canalicular networks and bone metabolism via the deregulation of the sclerostin and RANKL expression.

Osteoporosis is a major age-related disease. Intrinsic factors that determine the risk of osteoporosis and fractures include aging in addition to genetic, hormonal and metabolic factors. The pathophysiology of osteoporosis is based on an imbalance between bone formation by osteoblasts and bone resorption by osteoclasts^1^. Osteocytes, which represent approximately 95% of all bone cells, are embedded in the bone matrix and communicate via their dendritic processes with each other as well as bone surface cells, such as osteoblasts and osteoclasts, and vasculature cells^2^. Recently, osteocytes have also been shown to secrete soluble factors to target cells on both the bone surface and target distant organs, such as the kidneys, muscle and other tissues^2^. Furthermore, osteocytes exhibit numerous functions, including acting as orchestrators of bone remodeling by regulating both the osteoclast and osteoblast activity. Therefore, osteocytes play a variety of physiological roles in bone metabolism. However, the number of viable osteocytes in the human femur declines in an age-dependent manner, with the proportion of viable cells decreasing from 88% at 10–29 years of age to 58% at 70–89 years of age^3^,^4^. Furthermore, osteocyte cell death increases progressively with age in mice^2^. In addition, Milovanovic *et al.* reported that deterioration of the canalicular network with age reduces connectivity between osteocytes as well as other tissues^5^. However, the pathological mechanisms underlying the development of osteocyte dysfunction associated with aging remain poorly defined.

Accumulating evidence suggests that bone aging is associated with oxidative damage in both humans and animal models^6^,^7^,^8^,^9^. Oxidative damage is caused by an imbalance between the production of reactive oxygen species (ROS) and the clearance of ROS by antioxidant systems. As superoxide anion (O_2_^.−^), an ROS, is primarily generated as a metabolite of O_2_^.−^ during mitochondrial respiration^10^, we focused on the actions of mitochondrial superoxide dismutase 2 (SOD2), which is distributed in mitochondria, where it is metabolized from O_2_^.−^ to H_2_O_2._ *Sod2* deficiency has been reported to result in the upregulation of mitochondrial O_2_^.−^ with consequent oxidative damage to cells and tissues^11^,^12^,^13^,^14^. However, the pathological effects of *Sod2* deficiency on osteocytes have not been elucidated to date.

In the present study, we analyzed the bone phenotypes of *Sod2* conditional knockout mice with a disturbed mitochondrial redox balance and osteocyte function achieved via the Cre-loxP system under the control of the *Dmp1* promoter^15^,^16^. We herein discuss our findings indicating the pathological role of mitochondrial O_2_^.−^ present in osteocytes in bone metabolism.

## Results

### The aging process upregulates superoxide generation in osteocytes and impairs the osteocytic canalicular morphology

**Figure 1:**
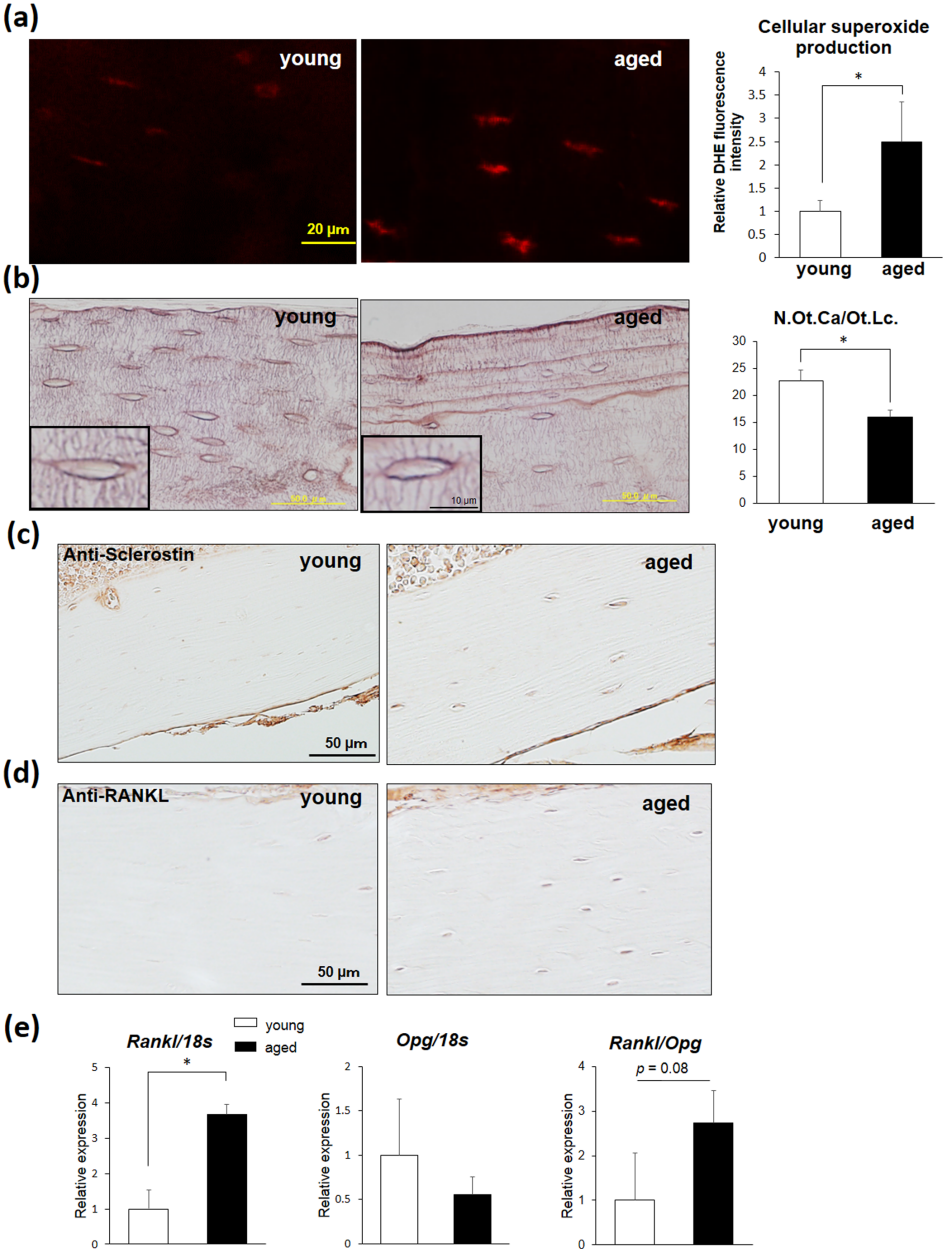
Superoxide generation is upregulated in osteocytes during aging. (a) Young (8 weeks of age) and aged (2 years of age) femurs were stained with dihydroethidium (DHE) (n = 3 each group). The scale bar indicates 20 μm. (b) AgNOR staining of osteocytic canaliculi in the endosteal region of the young (12 weeks of age) and aged (2 years of age) cortical femurs. The number of osteocyte canaliculi (N.Ot.Ca.) per osteocyte lacuna (Ot.Lc.) in the cortical femurs. The scale bars indicate 50 and 10 μm (inset), respectively. (c, d) Decalcified femoral sections were prepared from young (12 weeks of age) and aged (2 years of age) femurs and subjected to sclerostin and RANKL immunohistochemical staining. The scale bars indicate 50 μm. (e) The gene expression analysis of osteocyte-related genes in the flushed tibia obtained from young (12 weeks of age) and aged (2 years of age) mice. The data were normalized to the *18s* expression (n = 3–4 each group).

In order to evaluate the degree of oxidative damage in aged bone, we examined the level of O_2_^.−^ generation in aged cortical bone. Consequently, significant enhancement of O_2_^.−^ was observed in the aged osteocytes compared with that noted in the younger osteocytes (Fig. 1a). Furthermore, in order to investigate morphological changes in aged cortical bone, we performed AgNOR staining of cortical bone sections. The results showed a 29% reduction in the number of canaliculi per osteocyte lacuna (N.Ot.Ca/Ot.Lc.) in the aged bone at 2 years of age (16.00 ± 1.31 in aged vs 22.63 ± 1.99 in young; Fig. 1b). We next examined the distribution of sclerostin, a major negative regulator of bone formation, and RANKL, a major positive regulator of bone resorption, on femoral sections by an immunohistochemical technique. The analyses positively stained sclerostin and RANKL expression in the aged osteocytes compared to that observed in the young ones (Fig. 1c, d). Furthermore, gene expression analyses revealed a significant elevation of *Rankl* expression and *Rankl* and *Opg* ratio, suggesting that stress associated with aging alters the cellular superoxide levels in addition to the canalicular morphology and expression of osteoporosis-related genes (Fig. 1e).

### Generation of osteocyte-specific *Sod2*-deficient mice

In order to investigate the physiological and pathological roles of mitochondrial O_2_^.−^ in osteocytes, we generated conditional *Sod2*-deficient mice using the Cre-loxP system to induce mitochondrial O_2_^.−^. In addition, *Dmp1*-Cre transgenic mice were used to generate the selective expression of Cre proteins in late osteoblasts and osteocytes^15^,^16^. The subsequent crossbreeding of homozygous *Sod2*^flox/flox^ (wild-type) mice with the *Dmp1*-Cre transgenic mice gave rise to *Dmp1*-Cre, *Sod2*^flox/flox^ (*Sod2^ot/ot^*) mice. Alizarin red and Alcian blue staining showed no differences in skeletal formation with regard to the bone length or amount of cartilage between the *Sod2^ot/ot^* and wild-type neonates (Fig. S1a). Moreover, Western blot analyses demonstrated a specific loss of SOD2 proteins in the flushed tibia involving ablation of bone marrow cells in the *Sod2^ot/ot^* mice, whereas no such loss was observed in liver, brain or cardiac tissues (Fig. S1b).

### *Sod2* deletion in osteocytes increases cellular superoxide production

In order to isolate osteocytes from the mutant mice, we used CAG-CAT-EGFP reporter mice to induce the osteocyte-specific expression of EGFP in *Sod2^ot/ot^* mice. Consequently, we isolated the osteocyte-rich fraction obtained via the collagenase digestion of long bones in these mice using a high-purity isolation method^17^, then further gated EGFP^+^ cell populations from the fraction and analyzed the degree of mitochondrial and cellular O_2_^.−^ generation using MitoSox and DHE reagents, respectively (Fig, S1c). Both MitoSox and DHE staining revealed significant enhancement of the mitochondrial and cellular O_2_^.−^ levels in the mutant osteocytes. These results indicate that *Sod2* regulates the levels of cellular O_2_^.−^ in osteocytes.

### *Sod2* deficiency in osteocytes induces remarkable bone loss in an age-dependent manner

**Figure 2:**
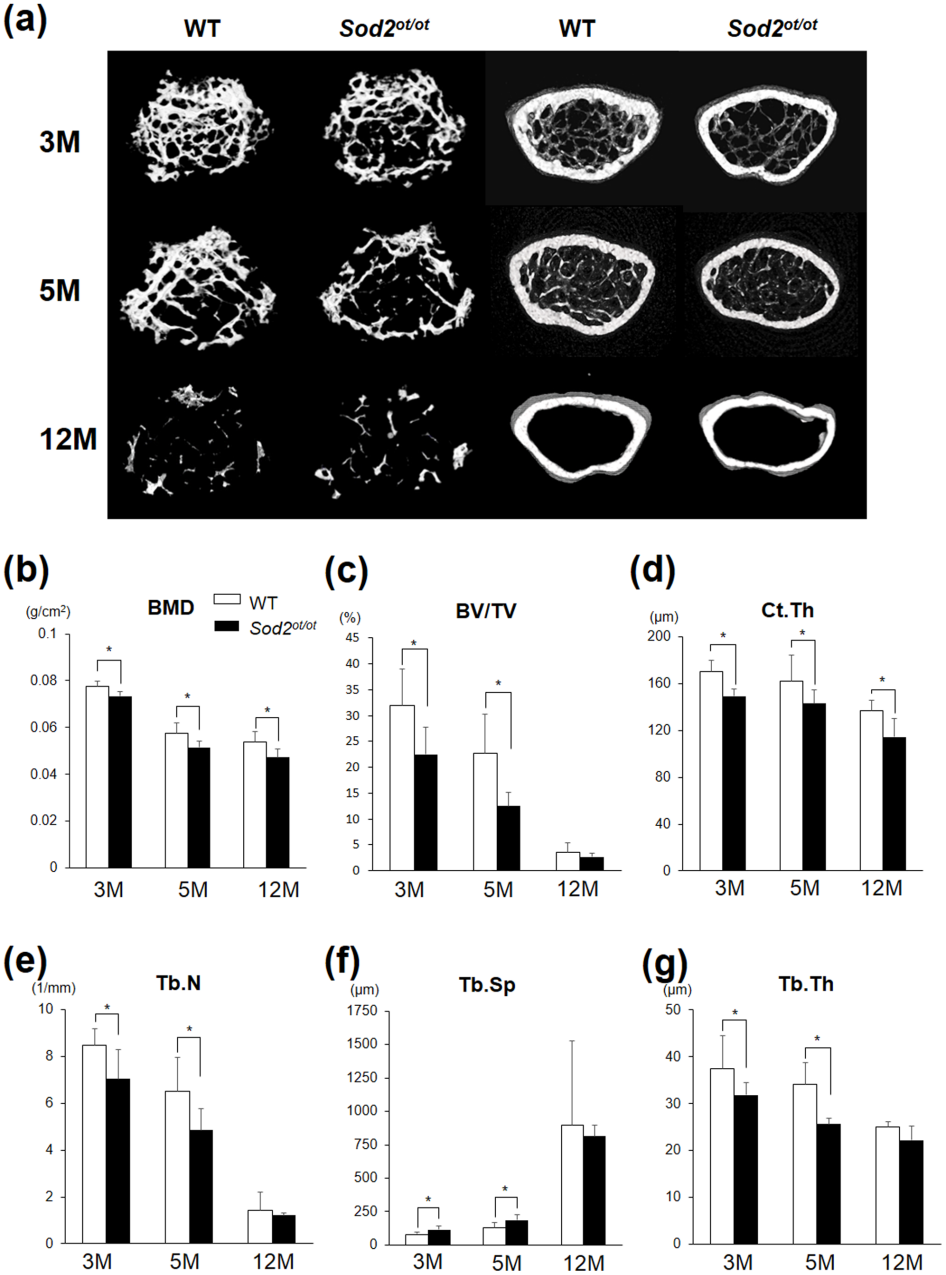
*Sod2* deficiency in osteocytes induces osteoporosis in an age-dependent manner. (a) 3D-μCT images of coronal sections of the femurs of the *Sod2^ot/ot^* and wild-type littermates at 3, 5 and 12 months of age (n = 5–6 each group). (b) The femoral BMD of the *Sod2^ot/ot^* and wild-type males at 3, 5 and 12 months of age (n = 5 each group). (c–g) The morphological parameters for the trabecular and cortical regions of the femurs at 3, 5 and 12 months of age measured according to μCT (n = 5–6 each group). (c) The bone volume per total volume (BV/TV), (d) cortical thickness (Ct.Th), (e) trabecular number (Tb.N), (f) trabecular separation (Tb.Sp) and (g) trabecular thickness (Tb.Th). *p < 0.05. The error bars indicate the SD.

In order to investigate the pathological effects of mitochondrial O_2_^.−^ on osteocytes, we investigated the skeletal phenotypes of *Sod2^ot/ot^* mice under conditions of aging. As a result, no differences were observed in body weight or femur length, indicating that endochondral ossification was not impaired in the mutant mice (Fig. S1d, e). In addition, we found that the femoral BMD was significantly decreased in the *Sod2^ot/ot^* mice compared with that observed in the wild-type mice under conditions of aging (Fig. 2b). A three-point bending analysis revealed significant fragility in the *Sod2^ot/ot^* femurs (Fig. S1f), while 3D-μCT analyses showed a marked decrease in the bone volume per tissues volume (BV/TV; Fig. 2a, c), cortical thickness (Ct.Th; Fig. 2d), trabecular number (Tb.N; Fig. 2e) and trabecular thickness (Tb.Th; Fig. 2g) in the *Sod2^ot/ot^* mice in an age-dependent manner. Likewise, we found a significant increase in trabecular separation (Tb.Sp; Fig. 2f) in the mutant mice. These results demonstrate that *Sod2* deficiency in osteocytes produces typical morphological signs of age-related osteoporosis.

### *Sod2* ablation impairs the canaliculi structure and survival of osteocytes

**Figure 3:**
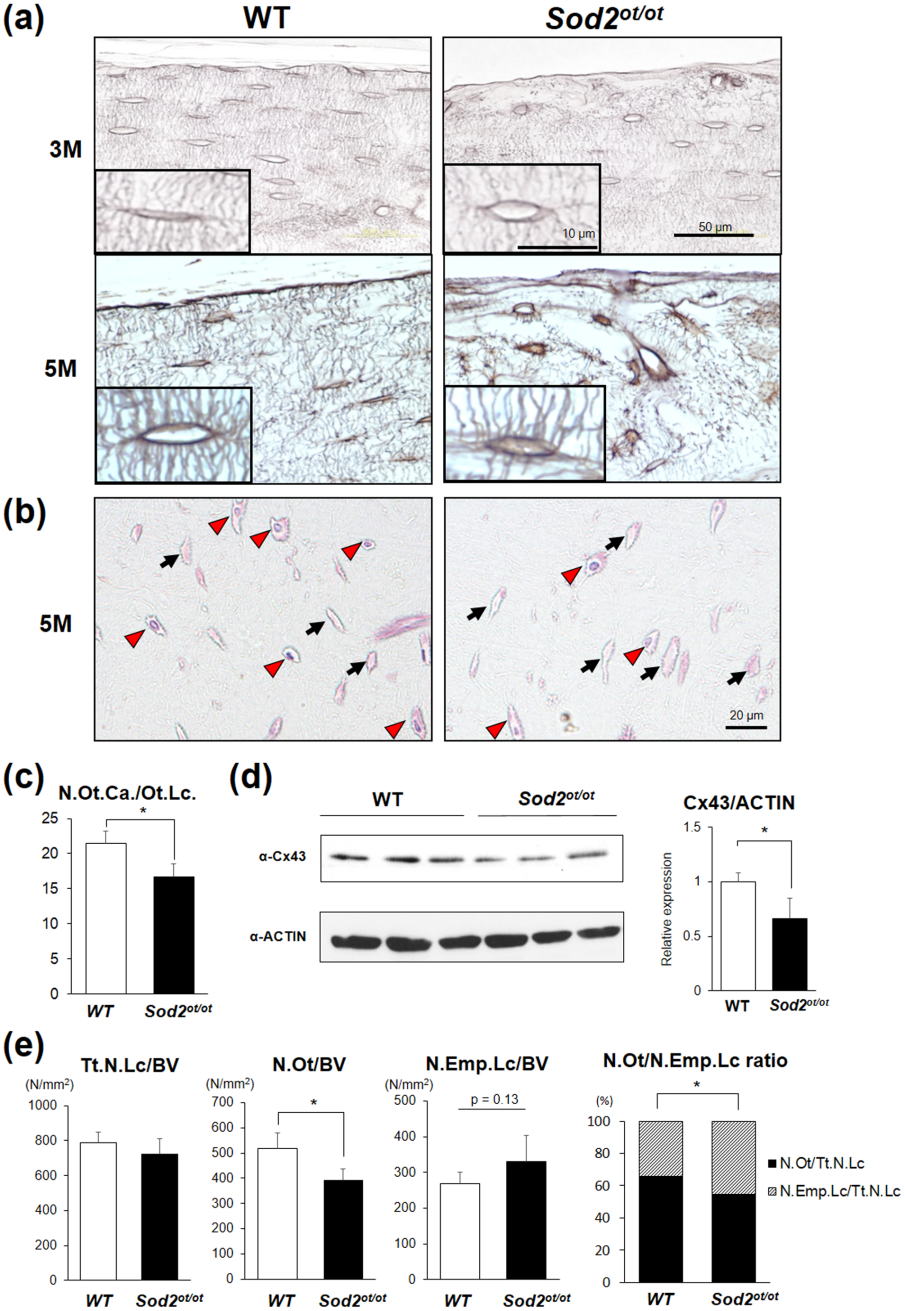
*Sod2* deletion in osteocytes impairs the canaliculi structure and osteocyte survival. (a) AgNOR staining of osteocytic canaliculi in the endosteal region of the cortical femurs of the *Sod2^ot/ot^* and wild-type male mice at 3 and 5 months of age. The scale bars indicate 50 and 10 μm (inset), respectively. (b) HE staining of femoral sections of the *Sod2^ot/ot^* and wild-type male mice at 5 months of age. The red arrowheads and black arrows indicate osteocyte lacunae and empty lacunae, respectively. The scale bar indicates 20 μm. (c) The number of osteocyte canaliculi (N.Ot.Ca.) per osteocyte lacuna (Ot.Lc.) in the cortical femurs (n = 4–5 each group). (d) A Western blot analysis of the Cx43 levels in the flushed tibia at 5 months of age. (e) The total number of lacunae per bone volume (Tt.N.Lc/BV), number of osteocyte lacunae per bone volume (N.Ot/BV), number of empty lacunae per bone volume (N.Emp.Lc/BV) and ratio of the number of osteocyte lacunae (N.Ot) to the number of empty lacunae (N.Emp.Lc) in the femoral cortical bone of the *Sod2^ot/ot^* and wild-type male mice at 5 months of age (n = 4–5 each group). *p < 0.05. The error bars indicate the SD.

In order to investigate morphological changes in *Sod2*-deficient osteocytes, we performed AgNOR staining of cortical bone sections. Interestingly, the *Sod2^ot/ot^* mice exhibited an abnormal canaliculi structure and density in an age-dependent manner (Fig. 3a). We also noted a 23% reduction in the number of canaliculi per osteocyte lacuna (N.Ot.Ca/Ot.Lc.) in the mutant bone at 5 months of age (16.63 ± 1.86 in *Sod2^ot/ot^* vs 21.44 ± 1.75 in wild-type; p < 0.001; Fig. 3a, b, c), recapitulating the N.Ot.Ca/Ot.Lc. values obtained in cortical bone specimens of young versus elderly humans^5^ and mice (Fig. 1b). Notably, *Sod2* loss also downregulated the expression of gap junction Cx43 proteins in the mutant bones (Fig. 3d), suggesting the pathological link between Cx43 downregulation and impairment of osteocytic canalicular networks. Next, we examined the number of osteocytes in the mutant bones using a histomorphometric analysis. Although the total number of lacunae (Tt.N.Lc/BV) did not differ in the *Sod2^ot/ot^* mice, the number of osteocytes (N.Ot/BV) was significantly decreased, while that of empty lacunae (N.Em.Lc/BV) was increased, in the cortical bone of the *Sod2^ot/ot^* femurs (Fig. 3e). Furthermore, the osteocyte versus empty lacuna ratio (N.Ot vs N.Emp.Lc ratio) was significantly decreased in the *Sod2^ot/ot^* cortical bones (Fig. 3e). Indeed, a scanning electron microscope (SEM) analysis revealed that the *Sod2^ot/ot^* mice had typical empty lacunae lesions at 3 months of age (Fig. S2). These results indicate that *Sod2* ablation alters the canalicular structure and cell fate of osteocytes.

### *Sod2* loss in osteocytes upregulates the sclerostin expression resulting in suppressed bone formation

**Figure 4:**
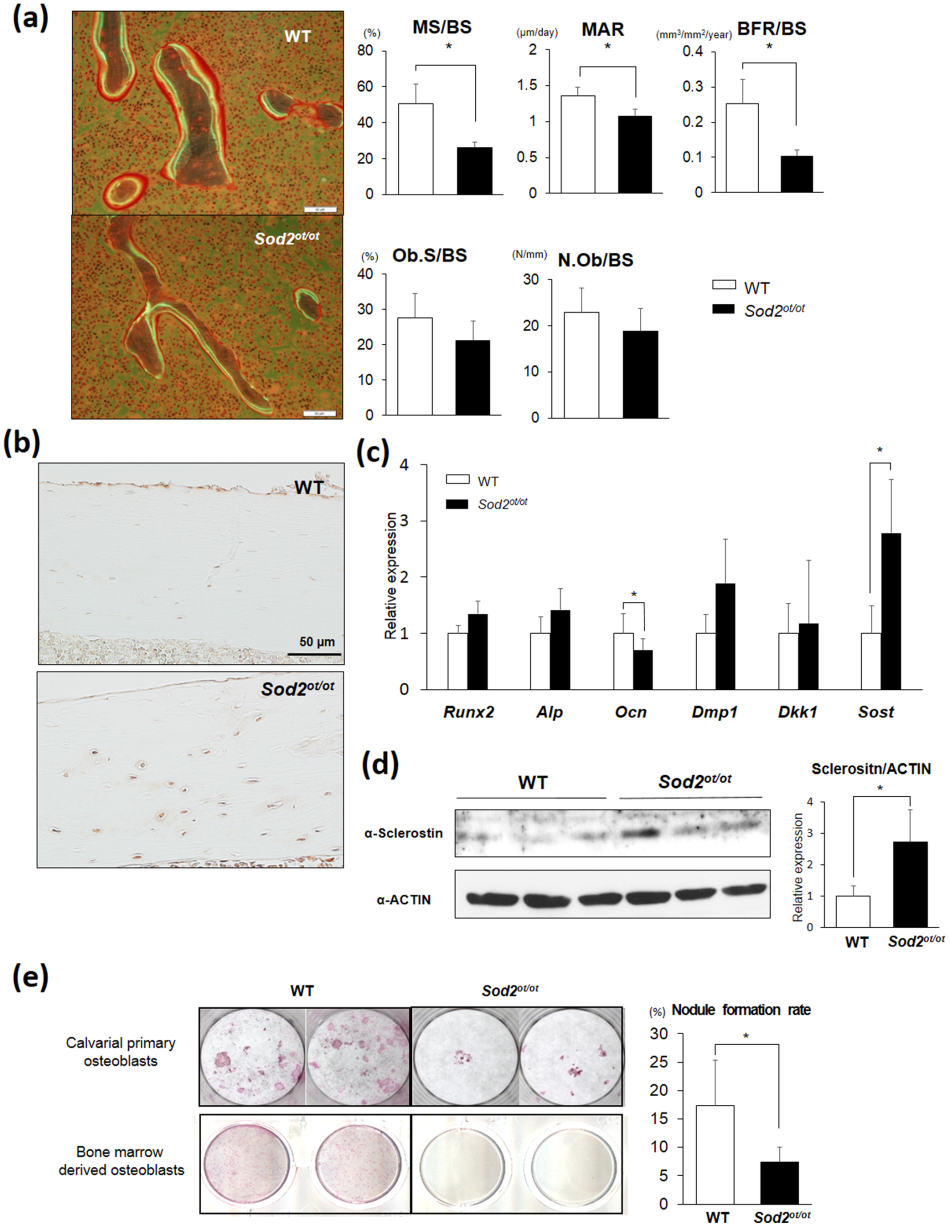
*Sod2* loss in osteocytes decreases the bone-forming activity associated with the upregulation of sclerostin. (a) Fluorescent micrographs of representative calcein-double labeling of *Sod2^ot/ot^* and wild-type male femurs at 5 months of age. The mineralized surface per bone surface (MS/BS), mineral apposition rate (MAR), bone formation rate (BFR/BS), osteoblast surface per bone surface (Ob.S/BS) and number of osteoblast per bone surface (N.Ob/BS) (n = 4–5 each group). (b) Decalcified femoral sections were prepared from *Sod2^ot/ot^* and wild-type male femurs at 5 months of age and subjected to sclerostin immunohistochemical staining. (c) A gene expression analysis of osteoblast- and osteocyte-related genes in the flushed tibia at 5 months of age. The data were normalized to the *Gapdh* expression (n = 5 each group). (d) A Western blot analysis of the sclerostin levels in the flushed tibia at 5 months of age using the same gel as in Figure 3d. (e) Mineralized nodule formation in the culture of primary bone-forming cells derived from neonatal calvaria (upper panel) and bone marrow (lower panel) on culture day 28. *p < 0.05. The error bars indicate the SD. The scale bar indicates 50 μm.

Next, we analyzed the bone remodeling state of the *Sod2^ot/ot^* mice using a calcein double labeling analysis. Although the osteoblast surface per bone surface (Ob.S/BS) and number of osteoblasts per bone surface (N.Ob/BS) did not differ in the mutant mice, the *Sod2^ot/ot^* mice displayed a significant decrease in the mineralizing surface per bone surface (MS/BS), mineral apposition rate (MAR) and bone formation rate per surface (BFR/BS), suggesting that SOD2 loss in osteocytes reduces the activity of bone formation (Fig. 4a, Tables S1 and S2). We next examined the distribution of sclerostin expression on femoral sections by an immunohistochemical technique. The analysis preferentially stained sclerostin expression in the *Sod2^ot/ot^* osteocytes compared to that observed in the wild-type ones (Fig. 4b). Notably, a qPCR analysis revealed that *Sod2* loss upregulated the expression of *Sost* and downregulated the expression of *Osteocalcin* in the *Sod2^ot/ot^* flushed tibias (Fig. 4c). We also found the sclerostin levels to be significantly increased in the *Sod2^ot/ot^* mice compared to that observed in the wild-type mice (Fig. 4d). To confirm whether mitochondrial O_2_^.−^ mediates *Sost* expression, we cultured osteocytic MLO-Y4 cells with paraquat, a mitochondrial O_2_^.−^ inducer at complex I^18^. We found that paraquat treatment significantly upregulated the *Sost* mRNA in the osteocytic cells (Fig. S3). In addition, the *in vitro* experiments revealed that *Sod2* deletion significantly suppressed mineralized nodule formation in primary bone-forming cells, including osteoblasts and osteocytes, derived from calvaria or bone marrow on culture day 28 (Fig. 4e). We further analyzed the cellular phenotypes of tamoxifen-induced *Sod2*-deficient bone-forming cells obtained from the calvaria of *Rosa26*-Cre^ERT2^ and *Sod2^flox/flox^* neonates. Consequently, treatment with tamoxifen (4-OHT) effectively depleted SOD2 proteins, thus resulting in mitochondrial O_2_^.−^ elevation on culture day 3 (Fig. S4a). On culture day 7, the *Sod2*^−*/*−^ cells exhibited delayed growth (Fig. S4b), followed by impairment of the alkaline phosphatase (ALP) activity and nodule formation on culture days 14 and 21, respectively (Fig. S4c, d). Taken together, these findings suggest that *Sod2* insufficiency impairs the *Sost* expression and bone-forming ability in osteoblast-osteocyte lineage cells in both cell-nonautonomous and -autonomous manners.

### Mitochondrial superoxide produced by *Sod2* loss promotes the upregulation of the RANKL expression associated with a higher rate of bone resorption

**Figure 5:**
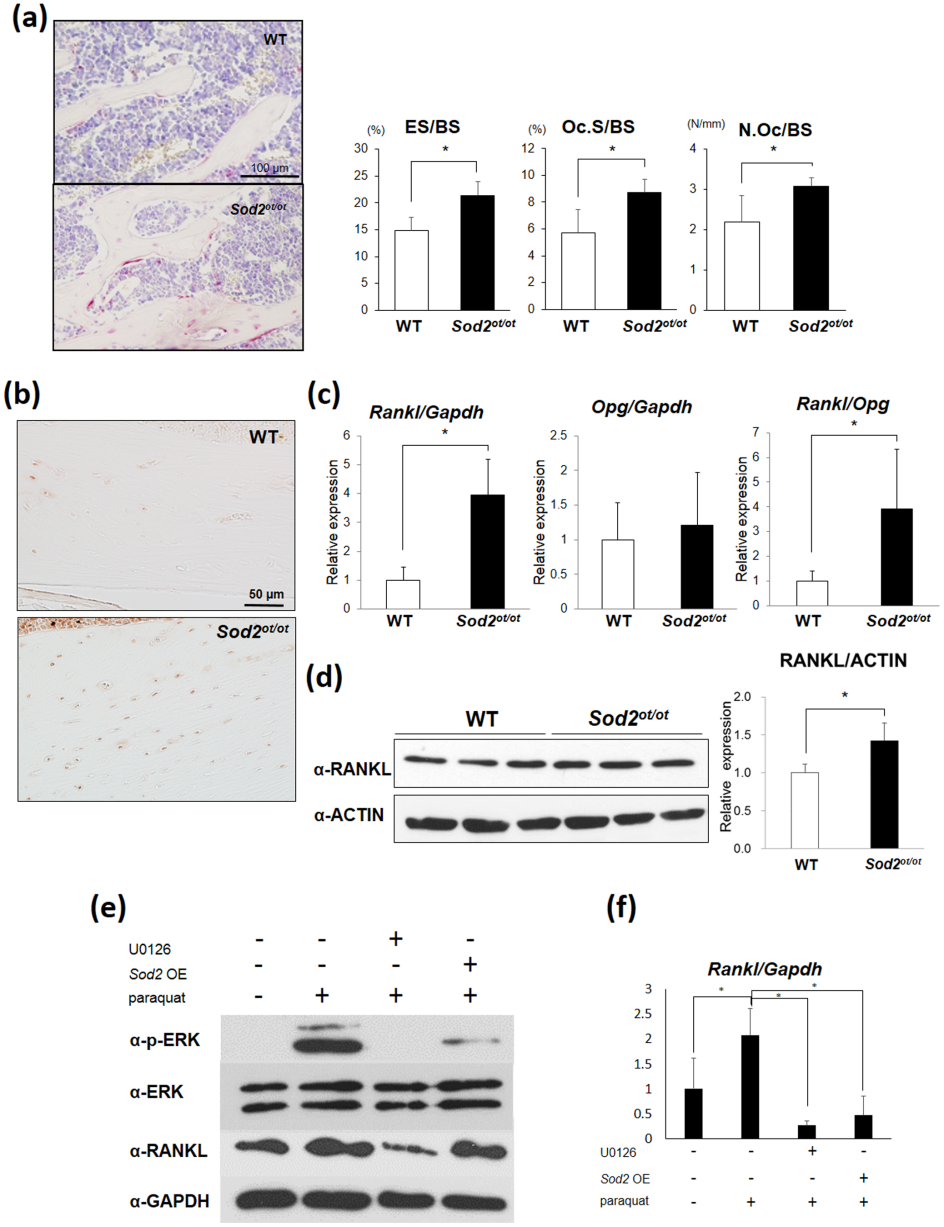
*Sod2* insufficiency in osteocytes increases bone resorption associated with the upregulation of RANKL. (a) Tartrate-resistant acid phosphatases (TRAP) staining of decalcified sections of the distal end of the femurs at 5 months of age (red signal). The eroded surface per bone surface (ES/BS), osteoclast surface per bone surface (Oc.S/BS) and number of osteoclast per bone surface (N.Oc/BS) (n = 5 each group). (b) Decalcified femoral sections were prepared from *Sod2^ot/ot^* and wild-type male femurs at 5 months of age and subjected to RANKL immunohistochemical staining. (c) A gene expression analysis of the *Rankl* and *Opg* genes in the flushed tibia at 5 months of age. The data were normalized to the *Gapdh* expression and represented as the *Rankl/Opg* ratio (n = 5 each group). (d) A Western blot analysis of the RANKL levels in the flushed tibia at 5 months of age using the same gel as in Figure 3d. (e) A Western blot analysis of RANKL and ERK phosphorylation in the MLO-Y4 cells treated with paraquat (1 mM) in the presence or absence of an ERK inhibitor (U0126) and *Sod2* overexpression for 24 hours. (f) A qPCR analysis of the *Rankl* expression in the MLO-Y4 cells treated with paraquat (1 mM) in the presence or absence of an ERK inhibitor (U0126) and *Sod2* overexpression for eight hours. *p < 0.05. The error bars indicate the SD. The scale bars indicate 100 μm (a) and 50 μm (b).

Next, we analyzed the bone resorption state of the *Sod2^ot/ot^* mice using a TRAP staining analysis. As a result, the histomorphometric analysis revealed that the *Sod2^ot/ot^* mice had increased eroded surface per bone surface (ES/BS), osteoclast per bone surface (N.Oc/BS) and osteoclast surface per bone surface (Oc.S/BS) values in the trabecular bone, indicating increased bone resorption (Fig. 5a). In order to investigate causes of the increase in bone resorption activity induced by SOD2 deficiency in the osteocytes, the distribution of RANKL expression on femoral sections was examined. The immunohistochemical analysis preferentially stained RANKL expression in the *Sod2^ot/ot^* osteocytes compared to that observed in the wild-type ones (Fig. 5b). We next extracted total RNA from flushed tibia without bone marrow and analyzed the *Rankl* and *Opg* expression levels. Consequently, a qPCR analysis revealed that *Sod2* loss significantly upregulated *Rankl*, but not *Opg*, transcription, resulting in a higher *Rankl* to *Opg* ratio in the *Sod2^ot/ot^* mice (Fig. 5c). We also found the RANKL protein expression to be significantly increased in the *Sod2^ot/ot^* flushed tibia (Fig 5d). Next, in order to exclude the presence of cell autonomous effects in the *Sod2^ot/ot^* osteoclasts, we cultured bone marrow cells with PTH and vitamin D to differentiate osteoclasts. Importantly, there were no changes in the capacity for osteoclast differentiation in the *Sod2^ot/ot^* mice compared with the wild-type mice (Fig. S5a). These findings indicate that *Sod2* deficiency in osteocytes increases the resorption activity via an increased RANKL expression.

In order to confirm whether mitochondrial O_2_^.−^ upregulates the *Rankl* expression in osteocytes, we cultured osteocytic MLO-Y4 cells with paraquat, an O_2_^.−^ generator in mitochondria. Notably, we found that paraquat treatment significantly enhanced the *Rankl* mRNA and protein expression levels in the MLO-Y4 cells (Fig. 5e, f). Conversely, *Sod2* overexpression completely suppressed *Rankl* upregulation in the presence of paraquat. Bai *et al.* previously reported that H_2_O_2_ stimulates the RANKL expression via the ERK and PKA-CREB pathways in mouse osteoblasts^19^. In this context, we analyzed the response of the ERK pathway to paraquat treatment in the osteocytes. Consequently, we found that paraquat treatment promoted the phosphorylation of ERK and that an ERK inhibitor (U0126) remarkably inhibited the RANKL upregulation induced by paraquat. These results suggest that mitochondrial O_2_^.−^ regulates the RANKL expression via, in part, the ERK pathway.

## Discussion

In the present study, we documented increased O_2_^.−^ generation and an altered canalicular structure in aged bone (Fig. 1). Our findings also demonstrated that increased mitochondrial O_2_^.−^ in osteocytes induced by *Sod2* depletion causes typical age-related osteoporosis associated with reduced bone formation and increased bone resorption (Figs. 4, 5). Interestingly, the *Sod2^ot/ot^* mice exhibited an abnormal canaliculi structure and osteocyte loss, resulting in the impairment of osteocytic canalicular networks (Fig. 3). Indeed, *Sod2* loss altered the gene expression of several bone-related genes, including *Cx43*, *Sost* and *Rankl*. Taken together, the present findings indicate that the mitochondrial O_2_^.−^ present in osteocytes regulates the cellular function and canalicular structure in the process of bone remodeling during aging.

Increased cellular O_2_^.−^ oxidizes cellular components, thereby inducing oxidative damage in tissues. However, whether an imbalance in O_2_^.−^ deregulates the cellular function in bone tissues has not been fully determined. Epidemiological studies have revealed a relationship between oxidative damage and osteoporosis in humans. In addition, Basu *et al.* reported that the urinary levels of 8-iso-prostaglandin F2a (8-iso-PGF2a, a major F2-isoprostane and biomarker of oxidative damage) are negatively associated with BMD and quantitative ultrasound measurements in humans^20^, and Maggio *et al.* found a marked decrease in the levels of plasma antioxidants, such as vitamins and SOD, in elderly osteoporotic females^21^. In animal studies, Almeida *et al*. demonstrated that chronological aging induces bone loss associated with increased levels of ROS and 4-hydroxynonenel, a product of lipid peroxidation, in bone marrow cells in both males and females, resulting in a redox imbalance in bone tissues^6^,^7^. The authors also reported that estrogen attenuates oxidative damage and regulates the cell fate of osteoblasts via DNA binding-independent actions of the ERα^22^. In addition, we previously demonstrated that the systemic deficiency of copper/zinc superoxide dismutase (*Sod1*), which catalyzes cellular O_2_^.−^ in the cytoplasm, induces cellular O_2_^.−^ generation and bone fragility via reductions in bone mass and the impairment of bone quality in mice^8^. Recently, we also reported that *Sod1* plays a pivotal role in protecting against bone loss induced by unloading^23^. In the present study, we demonstrated that excess mitochondrial O_2_^.−^ induced by osteocyte-specific *Sod2* deletion results in significant bone loss and fragility due to an imbalance in bone remodeling. Recently, Imai *et al*. reported that stress associated with senescence activates the Rb pathway and inactivates the Foxo3a pathway via the actions of AKT, thus resulting in *Sod2* downregulation and redox imbalance^24^. Taken together with the present findings, aging, senescence, estrogen insufficiency and *Sods* loss collectively induce cellular redox imbalances leading to the impairment of bone homeostasis.

Osteocytes are distributed throughout the bone matrix within a network of lacunae and canaliculi^2^. Osteocytes also directly connect various cells on/in bone tissues via dendritic processes in canaliculi. Notably, osteocytes produce and secrete soluble factors, including sclerostin and RANKL, in order to communicate indirectly with bone-related cells. Busse *et al*. revealed that the number of viable osteocytes in the human femur declines in an age-dependent manner, with the proportion of viable cells decreasing from 88% at 10–29 years of age to 58% at 70–89 years of age^3^,^4^. Furthermore, scanning electron microscopy (SEM) analyses have revealed that canalicular networks are impaired during aging based on an acid etching technique^5^, suggesting that the integrity of the canalicular network influences bone quality and fragility. In the present report, we found a similar reduction in both canalicular density and number in aged murine (Fig. 1b) and *Sod2*-deficient (Fig. 3) osteocytes, supporting the hypothesis that aging and/or redox imbalances in osteocytes commonly exacerbate the impairment of osteocytic canalicular networks and reduce survival in mammals.

Tatsumi *et al*. reported that targeted ablation of osteocytes using the 10-kb *Dmp1* promoter to drive the diphtheria toxin receptor expression in osteocytes eliminates approximately 70% of osteocytes in cortical bone and induces *Rankl*-induced osteoclast activation in mice^25^. In addition, this ablation model shows *Rankl* upregulation and *Sost* downregulation associated with the loss of bone mass and mechanosensing. In contrast, mechanical unloading via tail suspension induces the loss of bone mass and canalicular networks accompanied by *Rankl* and *Sost* upregulation. Interestingly, bed rest is associated with increased serum sclerostin levels in humans^26^. Our *Sod2^ot/ot^* mice showed similar bone phenotypes and molecular alterations, including abnormalities in the *Sost* and *Rankl* expression, to that observed in the unloading model, rather than the ablation model, suggesting the presence of a molecular link between O_2_^.−^ regulation and mechanosensing in osteocytes. Indeed, we previously reported that unloading induces cellular O_2_^.−^ generation in bone-forming cells, thus leading to acute bone loss^23^. Taken together, unloading and aging appear to induce redox imbalances resulting in impairment of the osteocyte function and communication in the setting of age-related osteoporosis.

With respect to morphological analyses, Chen *et al*. documented an abnormal mitochondrial structure and canalicular density in the osteocytes of aged senescence-associated mutant P6 (SAMP6) mice using an ultrastructural analysis, suggesting that senescence phenotypes affect the mitochondrial and canalicular structure in osteocytes^27^. In addition, we previously demonstrated that *Sod2* loss in cardiomyocytes results in the formation of abnormal mitochondria with swollen cristae exhibiting a decreased membrane potential and ATP production^12^. Interestingly, *Sod2* deficiency also significantly downregulates the expression of Cx43 proteins in the heart^28^. Similarly, our present findings indicate that mitochondrial *Sod2* ablation decreases the Cx43 protein levels in bone (Fig. 3) and bone-forming cells (data not shown), suggesting that *Sod2* loss generally suppresses the Cx43 expression and mitochondrial function in tissues. Furthermore, Bonewald *et al*. reported that ATP is an essential regulatory factor released in response to sheer stress and the mechanosensing response in osteocytes^2^. Taken together with our data, mitochondrial O_2_^.−^ appears to control the mitochondrial function and ATP production in order to maintain the mechanosensing activity via Cx43-mediated ATP release in osteocytes.

The present findings showed decreased formation of mineralized nodules and an increased expression of *Sost*, a negative regulator of Wnt signaling, in bone-forming cells, indicating a reduction in bone formation via both cell autonomous and non-autonomous pathways (Fig. 4). Furthermore, we detected an increased RANKL expression in the mutant bones, suggesting an increase in the bone resorption activity (Fig. 5). Baek *et al*. previously showed that a high-fat diet induces lipid accumulation, inflammation and bone loss associated with TNF-α, sclerostin and RANKL upregulation in osteocytes^29^ and demonstrated that TNF-α treatment enhances the *Sost* expression via the NF-κB pathway in MLO-Y4 cells. Meanwhile, Bai *et al*. reported that ROS stimulates the RANKL expression via the ERK and PKA-CREB pathways in mouse osteoblasts^30^, and Takami *et al.* documented the calcium/protein kinase C signal in osteoblasts/stromal cells to be the third signal required to induce the *Rankl* mRNA expression^31^. Moreover, Sato *et al*. reported that treatment with LPS or IL-1 upregulates the *Rankl* expression in osteoblasts via the MyD88-PKC-ERK axis^19^. In addition, when environmental stressors induce injury in bone, apoptotic osteocytes release RANKL in order to recruit osteoclasts and reconstruct the injured tissue^32^. In the present *in vitro* experiments, we showed that paraquat treatment stimulates the mitochondrial O_2_^.−^ production, *Sost* and *Tnf-α* upregulation (Fig. S3), as well as RANKL expression via ERK signaling in osteocytes (Fig. 5). Taken together, inflammatory mediators, such as TNF-α, IL-1 and ROS, cooperatively promote the *Sost* and *Rankl* expression in bone-forming cells, particularly osteocytes, under conditions of metabolic abnormalities and aging.

Recently, various therapeutic agents for osteoporosis have been developed. For example, anti-RANKL antibodies inhibit the binding of RANKL to RANK, thereby decreasing osteoclastogenesis and the bone resorption of mature osteoclasts. This antibody appears to be a promising, highly effective and safe parenteral therapy, with good adherence, in cases of osteoporosis^33^. Anti-sclerostin antibodies have also recently been developed and shown to increase bone formation and decrease bone resorption in clinical trials involving osteoporosis patients^34^. These clinical findings, together with the present data, indicate that these agents may be useful therapeutic drugs for age-related osteoporosis.

In conclusion, we herein demonstrated, for the first time, that osteocyte-specific *Sod2* loss causes typical age-related osteoporosis and impairs both osteocyte canalicular networks and survival. The *Sod2* present in osteocytes regulates the transcription of key molecules, such as *Sost* and *Rankl*, in the process of bone metabolism. Therefore, *Sod2* plays a pivotal role in the maintenance of bone homeostasis. Our findings suggest the potential of redox regulation in osteocytes as a therapeutic target for age-related and/or lifestyle-mediated osteoporosis.

## Methods

### Animals

The protocol for generating *Sod*2*^flox/flox^* mice has been previously described^11^. The mice were backcrossed with C57BL/6NCrSlc (Japan SLC, Shizuoka, Japan) mice for five or six generations. The crossbreeding of homozygous *Sod2^flox/flox^* mice (wild-type) with *Dmp1*-Cre transgenic mice^35^ gave rise to osteocyte-specific *Sod2*-deficient mice (*Sod2^ot/ot^*). All genotyping analyses of the *Dmp1*-Cre transgene and *Sod2^flox/flox^* mice were performed using PCR with genomic DNA isolated from the tail tip. In order to detect osteocytes in the mice, we generated osteocyte-specific GFP expressing-mice by crossbreeding CAG-CAT-EGFP reporter mice^36^ (Center for Animal Resources and Development, Kumamoto University, Japan) with *Dmp1*-Cre mice. The mice were maintained and studied according to protocols approved by the Animal Care Committee of Chiba University.

### Dual X-ray absorptiometry and microcomputed tomography analysis

The bone mineral density (BMD) of the isolated femur was measured using a PIXImus instrument (Lunar Corp., Madison, WI, USA). Microcomputed tomography scanning was performed with a ScanXmate-A090S Scanner (Comscantecno, Co., Ltd. Kanagawa, Japan). Three-dimensional microstructural image data were reconstructed and structural indices were calculated using the TRI/3D-BON software program (RATOC System Engineering, Kyoto, Japan). Bone morphometric analyses were performed at a region 0.3–0.6 mm above the distal growth plates in each femur. For cancellous bone, the bone volume/tissue volume (BV/TV), trabecular number (Tb. N), trabecular thickness (Tb. Th) and trabecular separation (Tb. Sp) values were measured. For cortical bone, the cortical thickness (Ct.Th) was measured.

### Bone strength test

The femurs were analyzed for bone stiffness using a bone strength tester (TK-252c, Muromachi Kikai, Tokyo, Japan).

### Histomorphometric analysis

The extent of superoxide generation in the femurs was measured in frozen sections of tissue, as previously described^37^. The femurs were stained with DHE solution (5 μM) for 30 minutes, and DHE labeling was visualized using a fluorescence microscope (Axiophoto, Zeiss, USA). The mineralized surface (MS/BS), mineral appositional rate (MAR) and bone formation rate (BFR/BS) in the cancellous regions of the femur were measured using undecalcified sections. The femurs of each mouse were removed and fixed with 70% ethanol, after which they were stained with Villanueva bone stain for six days, dehydrated in ascending grades of ethanol, defatted in an acetone/methyl-methacrylate monomer mixture (1:2) and embedded in methyl-methacrylate (Wako Chemicals, Kanagawa, Japan) without decalcification. Plastic blocks of Cross were cut with a precision bone saw in a cross direction along the femur. The blocks were mounted on plastic slides and cut, then ground to a thickness of 200 μm using a precision lapping machine (Maruto, Tokyo, Japan) and hand ground to a thickness of 15 ~ 19 μm according to the method of Frost^38^. We analyzed the calcein fluorescence-labeled double parallel expression in five or more locations in cancellous bone and calculated the average value. In the femoral cortical section, we counted the total number of lacunae per bone volume (Tt.N.Lc/BV), number of osteocyte lacunae per bone volume (N.Ot/BV), number of empty lacunae per bone volume (N.Emp.Lc/BV) and ratio of the number of osteocyte lacunae (N.Ot) to the number of empty lacunae (N.Emp.Lc). In the decalcified sections, tartrate-resistant acid phosphatase (TRAP)-positive multinucleated cells were counted as osteoclasts in order to determine the osteoclast surface/bone surface (Oc.S/BS) and osteoclast number/bone surface (N.Oc/BS) values. For the canaliculi structure analysis, the bone sections were incubated for 55 minutes at room temperature in the dark in AgNOR staining solution prepared by combining silver nitrate (2 volumes of 50% aqueous solution) and formic acid (1 volume of 1% solution containing 2% gelatin). After staining, the sections were thoroughly washed in distilled water and transferred for 10 minutes to a 5% aqueous sodium thiosulfate solution prepared extemporaneously. The sections were then rinsed with distilled water, mounted and observed using light microscopy. We counted the number of canaliculi per osteocyte lacuna (N.Ot.Ca/Ot.Lc.). Cytological smears were counterstained with nuclear fast red, dehydrated in ethanol and mounted in Entellan.

### Immunohistochemistry

Femur specimens from mice were fixed with 4% paraformaldehyde, decalcified with 10% EDTA and embedded in paraffin. Sections were deparaffinized, treated with 3% H_2_O_2_ to inhibit endogenous peroxidase activity, blocked with goat serum, and then incubated for overnight at 4°C with 1:10 dilution of the rabbit polyclonal anti-mouse sclerostin antibody (Sigma-aldrich) and 1:50 dilution of the rabbit polyclonal anti-mouse RANKL antibody (abcam). Sections then were incubated for 30 minute at room temperature with a 1:300 dilution biotinylated anti-rabbit IgG secondary antibody (Vector). Sections were further incubated for 30 minutes with a 1:300 dilution of peroxidase-conjugated streptavidin (DAKO) in 2% goat serum and developed with a DAB substrate-chromogen system (Dako) for up to 5 minutes. We confirmed no non-specific immunostaining with a rabbit control IgG as a primary antibody.

### MLO-Y4 cell culture

MLO-Y4 cells, a murine osteocyte-like cell line, were maintained in alpha modified Eagle's medium (α-MEM; Invitrogen, Carlsbad, CA, USA) supplemented with 2.5% fetal bovine serum and 2.5% calf serum (Hyclone, Logan, UT)^39^. Paraquat (1 mM) treatment was applied to the MLO-Y4 cells at eight hours for total RNA and 24 hours for protein. For the *Sod2* overexpression experiments, MLO-Y4 cells were transfected with pTA2 (TOYOBO) plasmid DNA for human *Sod2* using Lipofectamine 2000 (Invitrogen).

### Primary bone-forming cell culture

Cell suspensions resulting from primary isolation from neonatal calvaria or bone marrow were cultured on type-I rat tail collagen-coated six-well plates (Iwaki, Chiba, Japan) at a seeding density of approximately 250,000 cells per 9.5 cm^2^ in α-MEM supplemented with 10% FBS, 50 μg/ml of ascorbic acid (Sigma-Aldrich), 10 mM β-glycerophosphate (Sigma-Aldrich), 1% penicillin and 1% streptomycin (PS; CellGro, Manassas, VA, USA). The cells were maintained at 37°C and 5% CO_2_ in a humid-ified incubator. Mineralized nodule formation was measured with Calcified nodule Staining kit (COSMO BIO, Japan) at culture day 28.

### Western blot analysis

The tibia was thoroughly flushed bone marrow cells with saline. The flushed tibia was homogenized using a handy sonicator and then centrifuged at 12,000 × *g* for 30 minutes. The supernatant was subsequently assayed for the protein concentration using the DC Protein Assay Kit (BioRad, Hercules, CA). Equal amounts (20 μg) of total protein were subjected to 10% SDS-PAGE and electroblotted onto a PVDF membrane. The membranes were blocked in 2% blocking reagent (ECL Advance Blocking Agent, GE Healthcare) and probed with antibodies against Cx43 (1:1,000; Cell signaling), RANKL (1:1,000; abcam), SOD2 (1:2,000; SOD-110; StressGen), Sclerostin (1:500; abcam), p-ERK, ERK (1:1,000; Cell signaling), GAPDH (1:1,000; Cell signaling) and ACTIN (1:2,000; Cell signaling). The signals were detected using the ECL system (ECL plus, GE Healthcare) and a luminoimage analyzer LAS-3000 mini (Fuji Film, Tokyo, Japan).

### Real-time RT-PCR

The femur was thoroughly flushed bone marrow cells with saline. Total RNA was extracted from the flushed femur and MLO-Y4 cells using the Trizol reagent (Invitrogen), according to the manufacturer's instructions. cDNA was synthesized from 1 μg of total RNA using reverse transcriptase (RiverTraAce, TOYOBO). Real-time PCR was performed using a Mini Opticon (BIO-RAD) sequence detection system with the SYBR GREEN PCR Master Mix (BIO-RAD), according to the manufacturer's instructions. The detector was programmed with the following PCR conditions: 40 cycles of 15 seconds of denaturation at 95°C and 1 minute of amplification at 60°C. The results were normalized to the level of the housekeeping gene glyceraldehyde-3-phosphate dehydrogenase (*Gapdh*) and 18S ribosomal RNA. The relative differences in the PCR results were calculated using the comparative cycle threshold method. The following primer sets were used: *Gapdh* forward, 5′-AGAAGGTGGTGAAGCAGGCATC-3′ and reverse, 5′-CGAAGGTGGAAGAGTGGGAGTTG-3′. *18s* forward, 5′-GTAACCCGTTGAACCCCATT-3′ and reverse, 5′-CCATCCAATCGGTAGTAGCG-3′. *Sost* forward, 5′-TCCTGAGAAGAACCAGACCA-3′ and reverse, 5′-GCAGCTGTACTCGGACACATC-3′. *Rankl* forward, 5′-TGAAGACACACTACCTGACTCCTG-3′ and reverse, 5′-CCCACAATGTGTTTGCAGTTC-3′. *Opg* forward, 5′-TACCTGGAGATCGAATTCTGCTT-3′ and reverse, 5′-CCATCTGGACATTTTTTGCAAA-3′. *Runx2* forward, 5′-CCCAGCCACCTTTACCTACA-3′ and reverse, 5′-TATGGAGTGTGCTGCTGGTCTG-3′. *Alp* forward, 5′-GCTATCTGCCTTGCCTGTATCTG-3′ and reverse, 5′-AGGTGCTTTGGGAATCTGTGC-3′. *Ocn* forward, 5′-AAGCAGGAGGGCAATAAGGT-3′ and reverse, 5′-TAGGCGGTCTTCAAGCCATA-3′. *Dmp1* forward, 5′-CCCAGAGGGACAGGCAAATA-3′ and reverse, 5′-TCCTCCCCACTGTCCTTCTT-3′. *Dkk1* forward, 5′-CCGGGAACTACTGCAAAAAT-3′ and reverse, 5′-CCAAGGTTTTCAATGATGCTT-3′.

### Statistical analysis

The statistical analyses were performed using Student's *t-*test for comparisons between two groups. All data are expressed as the mean ± standard deviation (SD).

## Supplementary Material

Supplementary Information Supporting Information
