# Supplementary material for: Mitochondrial superoxide in osteocytes perturbs canalicular networks in the setting of age-related osteoporosis
Source: Sci Rep. 2015 Mar 16;5:9148. doi: 10.1038/srep09148 (PMC5376208; doi:10.1038/srep09148)
Supplement: Supplementary Information — Supporting Information [file srep09148-s1.pdf]

Supplementary Information

Mitochondrial superoxide in osteocytes perturbs canalicular networks in the setting of age-related osteoporosis

Keiji Kobayashi, Hidetoshi Nojiri, Yoshitomo Saita, Daichi Morikawa, Yusuke Ozawa, Kenji Watanabe, Masato Koike, Yoshinori Asou, Takuji Shirasawa, Koutaro Yokote, Kazuo Kaneko, and Takahiko Shimizu

Supplementary Figures S1-S6

Supplementary Table S1, S2

Supplementary Methods

Supplementary Reference

Supplementary Figures and Legends

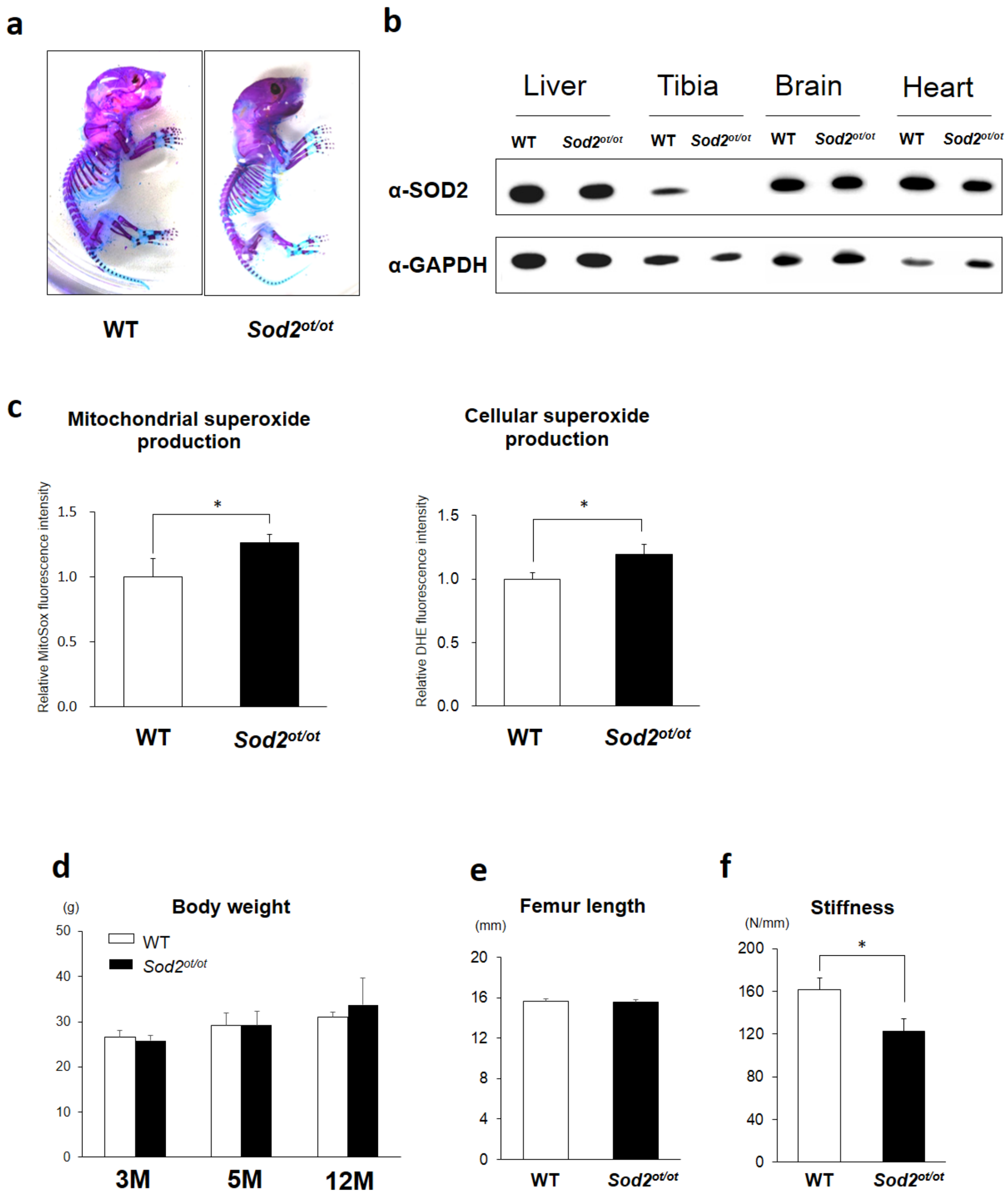

Supplementary Figure S1. Generation of osteocyte-specific *Sod2*-deficient mice. (a) Skeletal preparation of the *Sod2*$^{ot/ot}$ and wild-type neonates. (b) A Western blot analysis of the SOD2 proteins in the *Sod2*$^{ot/ot}$ and control mice. Protein extracts from the liver, flushed tibia, brain and heart of the *Sod2*$^{ot/ot}$ and wild-type mice were immunoblotted with anti-SOD2 and

anti-GAPDH antibodies. (c) Body weights of the *Sod2*$^{ot/ot}$ and wild-type littermates (n = 6 each group). (d) The length of the femur in the *Sod2*$^{ot/ot}$ and wild-type males at 3 months of age (n = 5 each group). (e) Bone stiffness in the male femoral bone at 3 months of age (n = 6 each group). (f) MitoSox (left) and DHE (right) staining for mitochondrial and cytoplasmic superoxide generation. *Sod2* loss in osteocytes derived from the CAG-CAT-EGFP, *Dmp1*-Cre, *Sod2*$^{\Delta/\Delta}$ mice (*Sod2*$^{ot/ot}$) resulted in enhanced superoxide production compared with that observed in the CAG-CAT-EGFP, *Dmp1*-Cre, *Sod2*$^{wt/wt}$ (WT) mice (n = 5-6 each group). *$p < 0.05$. The error bars indicate the SD.

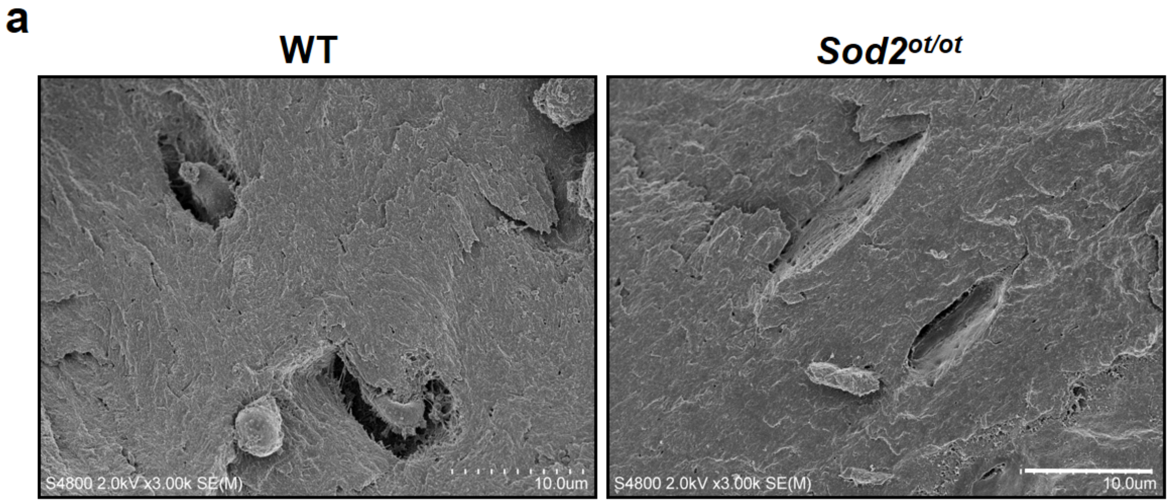

Supplementary Figure S2. *Sod2* deficiency in osteocytes increases the number of empty lacunae. (a) A scanning electron microscopic analysis of the cortical areas in the femurs of the *Sod2*$^{ot/ot}$ and wild-type littermates at 3 months of age. The scale bar indicates 10 μm.

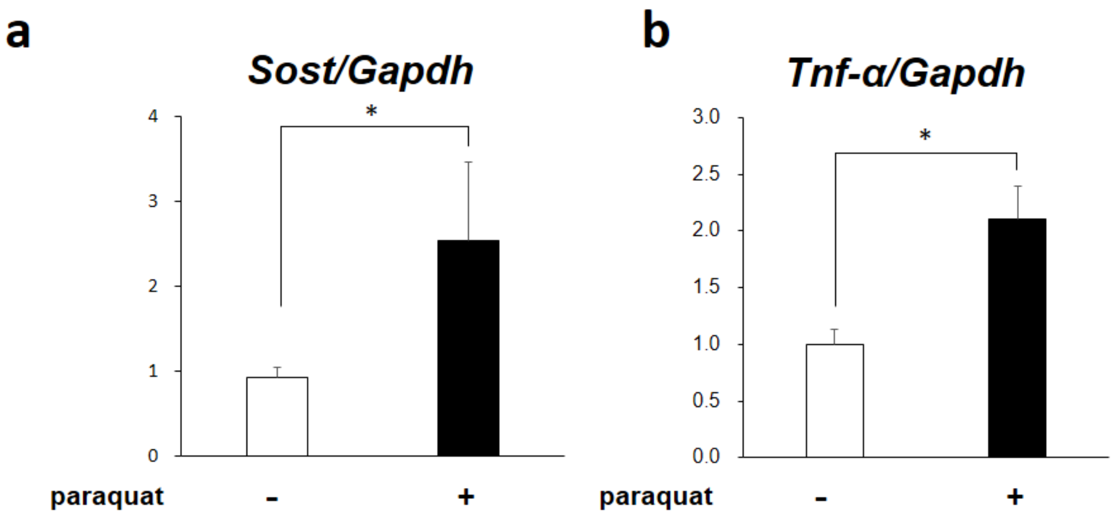

Supplementary Figure S3. (a) A gene expression analysis of the *Sost* expression in the MLO-Y4 cells treated with paraquat (1 mM) for eight hours. (b) A gene expression analysis of *Tnf-α* in the MLO-Y4 cells treated with paraquat (1 mM) for eight hours. *p < 0.05. The error bars indicate the SD.

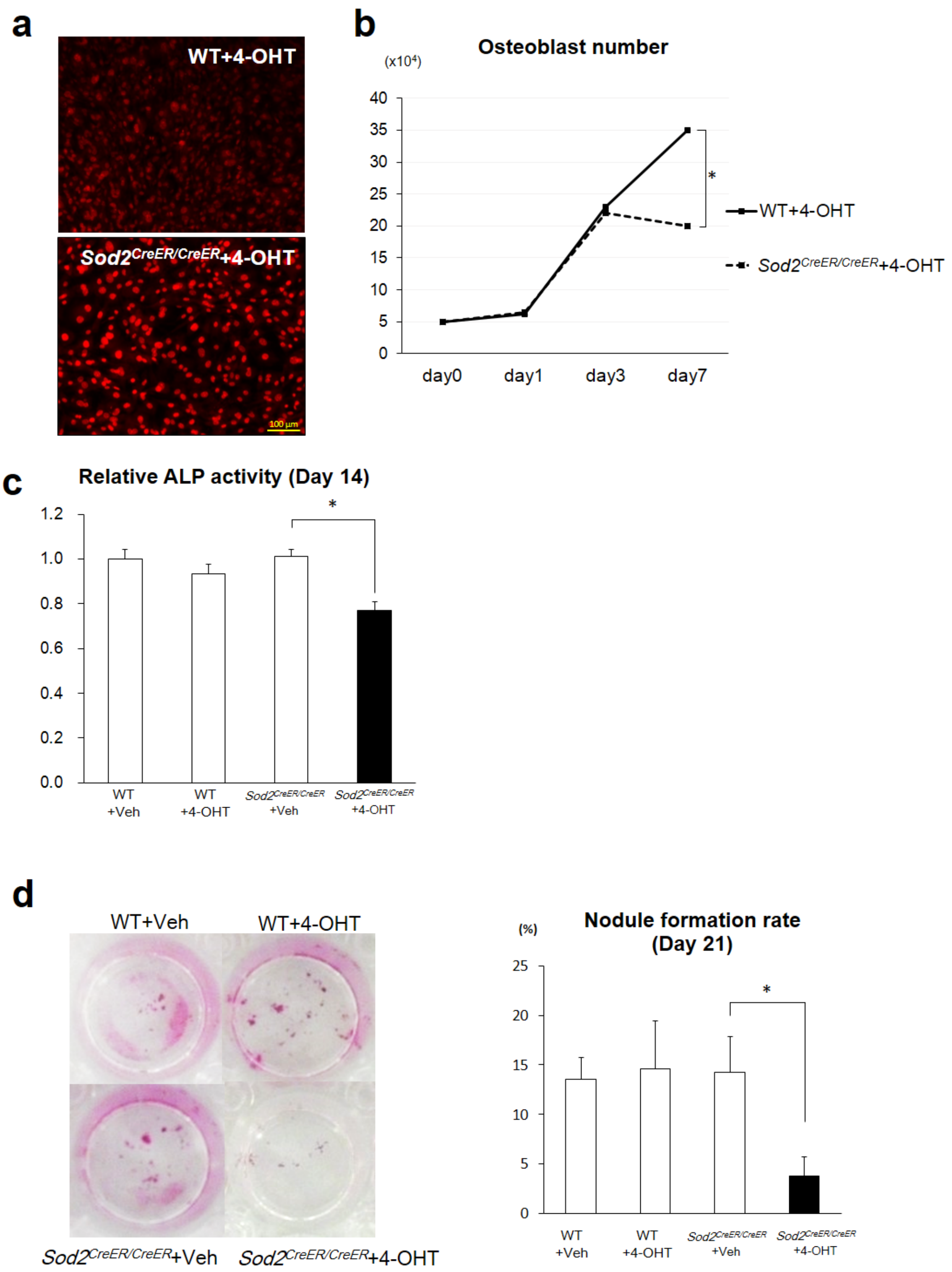

Supplementary Figure S4. *Sod2* deletion in bone-forming cells impairs cell growth and bone formation. (a) 4-hydroxytamoxifen (4-OHT) treatment induced the superoxide generation of

Rosa26-CreERT2 in the *Sod2*$^{\Delta/\Delta}$ (*Sod2*$^{CreER/CreER}$) and *Sod2*$^{f/f}$ (WT) mice using a DHE reagent on culture day 3. (b) Cell growth and (c) ALP activity on culture day 14 and (d) mineralized nodule formation on culture day 21 in the cultures of primary bone-forming cells derived from neonatal calvaria. *$p < 0.05$. The error bars indicate the SD. The scale bar indicates 100 μm.

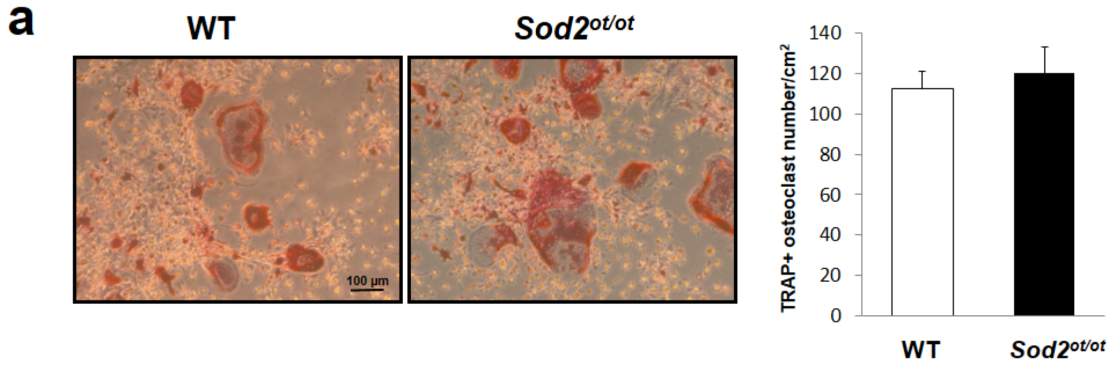

Supplementary Figure S5. Normal osteoclast differentiation from *Sod2$^{ot/ot}$* bone marrow. (a) In the *in vitro* osteoclast formation assay, bone marrow cells were cultured in α-MEM/10% FBS in the presence of 10 nM PTH and 10 nM 1α,25$(OH)_2$ vitamin D3 for seven days. The cells were fixed and stained with TRAP reagent. TRAP$^+$ multinucleated (> 3 nuclei) cells were counted as mature osteoclasts (n = 5 each group).

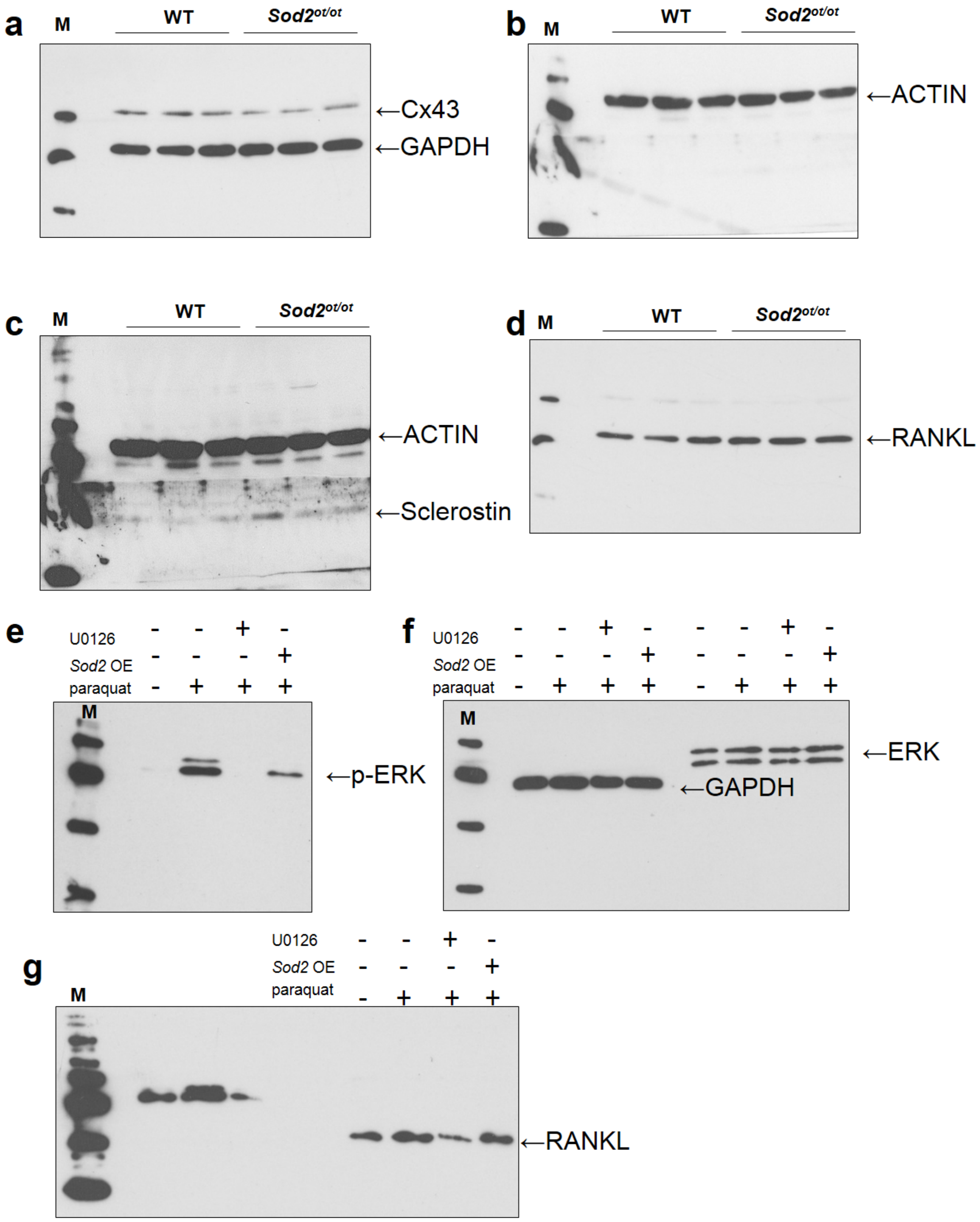

Supplementary Figure S6. Full-length images for Figure 3d, 4d, 5d and 5e. (a) Cx43 (Fig. 3d). (b) ACTIN (Fig. 3d, 4d and 5d). (c) sclerostin (Fig. 4d) (d) RANKL (Fig. 5d) (e) p-ERK (Fig. 5e) (f) GAPDH and ERK (Fig. 5e) (g) RANKL (Fig. 5e) for western blotting detection with anti-rabbit secondary antibodies. M: marker

Table S1. Trabecular bone morphological parameters of *Sod2*$^{ot/ot}$ and wild-type at 5 months of age.

		WT	Sod2$^{ot/ot}$	p value
BV/TV	%	13.94	9.96	0.027
OV/TV	%	0.58	0.32	0.111
OV/BV	%	4.16	3.09	0.320
Tb.Th	μm	38.92	28.68	0.001
OS/BS	%	35.55	25.21	0.090
Ob.S/OS	%	77.73	85.18	0.019
Ob.S/BS	%	27.61	21.23	0.145
ES/BS	%	14.82	21.31	0.004
Oc.S/ES	%	39.22	41.07	0.772
Oc.S/BS	%	5.72	8.73	0.010
N.Mu.Oc/BS	N/mm	1.27	2.10	0.007
N.Mu.Oc/ES	N/mm	8.77	9.88	0.507
N.Ob/BS	N/mm	22.84	18.81	0.244
N.Ob/OS	N/mm	64.68	75.54	0.005
N.Oc/BS	N/mm	2.18	3.07	0.022
N.Oc/ES	N/mm	15.00	14.54	0.855
N.Mu.Oc/TV	N/mm²	9.18	14.21	0.024
N.Oc/TV	N/mm²	15.78	21.26	0.151
N.Ob/TV	N/mm²	163.89	132.26	0.362
MAR	μm/day	1.36	1.07	0.007
BFR/BS	mm³/mm²/year	0.25	0.10	0.004
BFR/BV	%/year	1322.25	702.02	0.026
BFR/TV	%/year	179.60	77.77	0.006
BS/TV	μm/μm²	0.01	0.01	0.748
BS/BV	μm/μm²	0.05	0.07	0.000
dLS/BS	%	45.55	19.83	0.003
sLS/BS	%	10.03	12.98	0.163
Vd(d+s)LS/BS	%	5.34	7.27	0.171
LS/BS	%	55.58	32.82	0.004
MS/BS	%	50.57	26.33	0.004

BV: Bone Volume, BS: Bone Surface, TV: Tissue Volume, OV: Osteoid Volume, Tb.Th: Trabecular Thickness, OS: Osteoid Surface, Ob.S: Osteoblast Surface, ES: Eroded Surface, Oc.S: Osteoclast Surface, N.Mu.Oc: number of Multi-nuclear Osteoclast, N.Oc: Number of Osteoclast, MAR: Mineral Apposition Rate, BFR: Bone Formation Rate, dLS: double Labeling Surface, sLS: single Labeling Surface, MS: Mineralizing Surface.

Table S2. Cortical bone morphological parameters of *Sod2*$^{ot/ot}$ and wild-type at 5 months of age.

		WT	Sod2$^{ot/ot}$	p value
Ct.Ar	mm²	0.84	0.69	0.005
Ps.dLS/Ps(BS)	%	77.76	24.09	0.001
Ps.sLS/Ps(BS)	%	1.27	1.04	0.754
Ps.Vd(d+s)/Ps(BS)	%	12.10	12.63	0.934
Es.dLS/Es(BS)	%	46.18	31.62	0.087
Es.sLS/Es(BS)	%	5.12	5.93	0.680
Es.Vd(d+s)/Es(BS)	%	7.44	8.06	0.876
Ps.MAR	μm/day	1.60	0.84	0.026
Es.MAR	μm/day	1.09	0.78	0.070
Ps.BFR	μm²/day	6449.32	1355.42	0.003
Es.BFR	μm²/day	1916.44	1072.89	0.044
Ps.BFR/Ps(BS)	mm³/mm²/year	0.50	0.11	0.006
Es.BFR/Es(BS)	mm³/mm²/year	0.21	0.12	0.044
BFR/BS	mm³/mm²/year	0.71	0.23	0.005
BFR/Ct.Ar	%/year	369.43	130.61	0.010
Ct.Wi	mm	0.21	0.18	0.007
BFR/Ct.Ar	%/day	1.01	0.36	0.010
Ps.OS/Ps(BS)	%	94.92	45.63	0.001
Ps.ES/Ps(BS)	%	1.09	15.21	0.006
Ps.QS/Ps(BS)	%	4.00	39.16	0.001
Es.OS/Es(BS)	%	52.15	39.81	0.043
Es.ES/Es(BS)	%	20.92	15.83	0.229
Es.QS/Es(BS)	%	26.93	44.37	0.022
Po.Ar/Ct.Ar	%	0.15	0.12	0.610
MS/BS(Ps)	%	84.44	30.92	0.001
MS/BS(Es)	%	52.46	38.61	0.118
MS/BS（Ps+Es)	%	71.32	34.23	0.004

Ct.Ar: Cortical Area, BS: Bone Surface, ES: Eroded Surface, MAR: Mineral Apposition Rate, BFR: Bone Formation Rate, dLS: double Labeling Surface, sLS: single Labeling Surface, MS: Mineralizing Surface, Ps: Periosteal, Es: Endosteal, QS: Quiescence Surface, OS: Osteoid Surface.

Supplementary Methods

Skeletal preparation. Mice skeletons at 1 day after birth were fixed in 95% ethanol. Cartilage tissues of the specimens were stained with 0.2% Alcian Blue 8GX (Sigma) in ethanol-glacial acetic acid for 1 day and treated with 2% KOH for overnight. Mineralized bone was stained with Alizarin Red (Sigma) in 0.5% KOH, treated with a graded series of KOH-glycerol, and stored in glycerol.

Isolation of primary osteocytes. Osteocytes were isolated from mouse long bones utilizing a modified protocol derived from the method of Stern A.R. *et al.* {Stern, 2012 #8;Stern, 2012 #8}. Mice with an osteocyte-specific expression of EGFP were generated by crossbreeding CAG-CAT-EGFP with *Dmp1*-Cre, *Sod2*$^{\Delta/\Delta}$ or *Dmp1*-Cre, *Sod2*$^{wt/wt}$ transgenic mice. Using fluorescence-activated cell sorting (FACS Cant Ⅱ, BD, USA), EGFP-positive osteocytes were gated from the cell fractions (fractions 7-9) obtained via the sequential enzymatic digestion of long bones (femur, tibia and humeri). The bones from each individual mouse were pooled together and treated as one sample. Collagenase solution was prepared as 300 active U/mL collagenase type-IA (Sigma-Aldrich, St. Louis, MO, USA) dissolved in α-minimal essential medium (α-MEM). EDTA tetrasodium salt dehydrate solution (5 mM, pH 7.4; Dojindo) was prepared in phosphate-buffered solution (PBS; Takara) with 1% BSA (Sigma-Aldrich). Following each sequential digestion, the digest solution with suspended cells was removed from the bone pieces and kept. The bone pieces were then rinsed with Hank's balanced salt solution (HBSS; Invitrogen, Carlsbad, CA, USA) three times, and the rinsate was added to the digestion solution. The combined cell suspension solution was spun down at 200 x *g* for 5 minutes, after which the supernatant was removed from the cell pellets and the cells were resuspended in α-MEM supplemented with 10% fetal bovine serum (FBS; HyClone Laboratories, Logan, UT, USA) and counted. Isolated EGFP-positive osteocytes were stained

with 10 μM of MitoSox (Invitrogen, Carlsbad, CA, USA) and dihydroethidium (DHE, Invitrogen, Carlsbad, CA, USA) dyes for 30 minutes at 37°C. EGFP-positive osteocytes were assessed with respect to their cell number and O_2^- production using a flow cytometer (FACS CantⅡ, BD, USA). The data for the average intensity were analyzed using the FlowJo software program (TREE STAR, OR, USA).

Scanning electron microscopy (SEM) analysis. For the ultrastructural analysis, the specimens were immersed in a mixture of 2% paraformaldehyde and 2.5% glutaraldehyde in 0.067 M cacodylate buffer (pH 7.4), post fixed in a mixture of 1% osmium tetroxide and 1.5% potassium ferrocyanide and embedded in epoxy resin. Ultrathin sections were examined under an SEM (Hitachi S-800, Hitachi).

MLO-Y4 cell culture. MLO-Y4 cells, a murine osteocyte-like cell line, were maintained in alpha modified Eagle's medium (α-MEM; Invitrogen, Carlsbad, CA, USA) supplemented with 2.5% fetal bovine serum and 2.5% calf serum (Hyclone, Logan, UT). Paraquat (1 mM) treatment was applied to the MLO-Y4 cells at eight hours for total RNA.

Real-time RT-PCR. Total RNA was extracted from MLO-Y4 cells using the Trizol reagent (Invitrogen), according to the manufacturer's instructions. cDNA was synthesized from 1 μg of total RNA using reverse transcriptase (RiverTraAce, TOYOBO). Real-time PCR was performed using a Mini Opticon (BIO-RAD) sequence detection system with the SYBR GREEN PCR Master Mix (BIO-RAD), according to the manufacturer's instructions. The detector was programmed with the following PCR conditions: 40 cycles of 15 seconds of denaturation at 95°C and 1 minute of amplification at 60°C. The results were normalized to the level of the housekeeping gene glyceraldehyde-3-phosphate dehydrogenase (*Gapdh*)

ribosomal RNA. The relative differences in the PCR results were calculated using the comparative cycle threshold method. The following primer sets were used: *Gapdh* forward, 5'-AGAAGGTGGTGAAGCAGGCATC-3' and reverse, 5'-CGAAGGTGGAAGAGTGGGAGTTG-3'. *Sost* forward, 5'-TCCTGAGAAGAACCAGACCA-3' and reverse, 5'-GCAGCTGTACTCGGACACATC-3'. *Tnf-α* forward, 5'-ATGAGCACAGAAAGCATGATCCGC -3' and reverse, 5'-GCTTGGTGGTTTGCTACGAC -3'

Primary bone-forming cell culture. Cell suspensions resulting from primary isolation from neonatal calvaria of *Rosa26*-CreERT2[2] in the *Sod2*$^{\Delta/\Delta}$ were cultured on type-I rat tail collagen-coated six-well plates (Iwaki, Chiba, Japan) at a seeding density of approximately 250,000 cells per 9.5 cm^2 in α-MEM supplemented with 10% FBS, 50 μg/ml of ascorbic acid (Sigma-Aldrich), 10 mM β-glycerophosphate (Sigma-Aldrich), 1% penicillin and 1% streptomycin (PS; CellGro, Manassas, VA, USA). The cells were maintained at 37°C and 5% CO_2 in a humidified incubator. 4-hydroxytamoxifen (4-OHT) treatment induced the superoxide generation of Rosa-CreERT2 in the *Sod2*$^{\Delta/\Delta}$ (*Sod2*$^{CreER/CreER}$) and *Sod2*$^{f/f}$ (WT) mice using a 10μM DHE reagent for 30 minutes on culture day 3. ALP activities were measured on culture day 14 using TRACP & ALP Assay kit (TAKARA, JAPAN). Mineralized nodule formation was measured with Calcified nodule Staining kit (COSMO BIO, JAPAN) at culture day 21.

***In vitro* osteoclastogenesis.** Bone marrow cells derived from *Sod2*$^{ot/ot}$ and wild-type mice were seeded and cultured in α-MEM with 10% FBS containing 10 nM PTH (Sigma) and 10

nM 1α,25$(OH)_2$ vitamin D3 (Sigma). The cells were fixed and stained for TRAP. TRAP-positive multinucleated (> 3 nuclei) cells were counted as osteoclast-like cells.

Supplementary Reference

1. Stern, A.R.*, et al.* Isolation and culture of primary osteocytes from the long bones of skeletally mature and aged mice. *Biotechniques* **52**, 361-373 (2012).
2. Badea, T.C. *et al.* A noninvasive genetic/pharmacologic strategy for visualizing cell morphology and clonal relationships in the mouse. *J. Neurosci.* **23**, 2314 -2322 (2003).
